# Climate change attribution and legal contexts: evidence and the role of storylines

**DOI:** 10.1007/s10584-021-03177-y

**Published:** 2021-08-02

**Authors:** Elisabeth A. Lloyd, Theodore G. Shepherd

**Affiliations:** 1 Department of History and Philosophy of Science and Medicine, Indiana University, Bloomington, IN 47401 USA; 2 Department of Meteorology, University of Reading, Reading, RG6 6BB UK

**Keywords:** Climate change, Extreme event attribution, Causation, Climate change litigation, Climate change liability, Loss and damage

## Abstract

In a recent very influential court case, *Juliana v. United States*, climate scientist Kevin Trenberth used the “storyline” approach to extreme event attribution to argue that greenhouse warming had affected and will affect extreme events in their regions to such an extent that the plaintiffs already had been or will be harmed. The storyline approach to attribution is deterministic rather than probabilistic, taking certain factors as contingent and assessing the role of climate change conditional on those factors. The US Government’s opposing expert witness argued that Trenberth had failed to make his case because “all his conclusions of the injuries to Plaintiffs suffer from the same failure to connect his conditional approach to Plaintiffs’ local circumstances.” The issue is whether it is possible to make statements about individual events based on general knowledge. A similar question is sometimes debated within the climate science community. We argue here that proceeding from the general to the specific is a process of deduction and is an entirely legitimate form of scientific reasoning. We further argue that it is well aligned with the concept of legal evidence, much more so than the more usual inductive form of scientific reasoning, which proceeds from the specific to the general. This has implications for how attribution science can be used to support climate change litigation.

“The question is”, said Alice, “whether you can make words mean different things.” “The question is”, said Humpty Dumpty, “which is to be master — that’s all.” (Lewis Carroll, Alice’s Adventures in Wonderland).

## Introduction

Increasingly, claims concerning damage from anthropogenic climate change are reaching the courts, where they fall under tort law (Marjanac and Patton 2018; Burger et al. 2020). At the heart of liability under tort law is the concept of causality, for which expert testimony is crucial (Cranor 2005). Climate-related damage generally arises from extreme weather and climate events, whose causality is the subject of what is called extreme event attribution (NAS 2016). The question thus arises of how the science of extreme event attribution can be used to establish the causality of climate-event-related harms within the specific context of tort law.

In the recent case of *Juliana v. United States* (see, e.g., Burger et al. 2020, section III.C.3.b.i, and further discussion in Section 2), the plaintiffs claimed constitutional violations via the specific harms they had undergone because of damage from greenhouse gas induced global warming (for which the US government was deemed partly responsible) having aggravated particular kinds of extreme weather and climate events in the USA. Among the legal questions involved, there is an event attribution question at the heart of the case. Climate scientist Kevin Trenberth gave testimony using the causal, conditional, “storyline” attribution method (Shepherd 2016) supporting the plaintiffs. This approach takes certain factors as contingent and assesses the role of climate change conditional on those factors. The US Government used integrated assessment modeler John Weyant to rebut Trenberth’s analyses, essentially arguing that a fully quantitative modeling chain down to the scale of the specific harm, including the human-managed element, was necessary to establish causation. Climate lawyers Burger et al. (2020) explain why the Juliana case is so significant:

*Juliana* illustrates some of the challenges plaintiffs may face in establishing a causal connection to individual injuries… the plaintiffs dedicated a large portion of their briefs and expert testimony to defining that causal nexus between climate change and specific injuries, and if the case had gone to trial, this would have been one of the key factual disputes. One critical question for courts as they begin to grapple with such factual disputes is to what extent observational evidence of local impacts (e.g., loss of snowpack at ski resorts) can be used to support claims of injury in the absence of an attribution study of a matching geographic and temporal scope showing that the observed impact was caused by anthropogenic influence on climate change.

Weyant’s demand for an attribution study *of a matching geographic and temporal scope*, down to the scale of a specific ski resort or road or cabin, is clearly going to be difficult to achieve. Moreover, as Hulme et al. (2011) have argued, the connection between hazard (the weather or climate event) and impact or harm is “always mediated through complex political, social, and economic structures”, which would need to be considered in any assessment of liability (Lusk 2017). Thus, the question posed by Burger et al. (2020) is whether, given the challenges of performing such a detailed study in a comprehensive way, it is nevertheless possible to reason from the general to the specific, taking account of the specificity in a contingent, conditional manner. Were this possibility to be excluded, it would largely preclude the use of tort law to address climate damage from individual extreme events. Because *Juliana* has now been dismissed on unrelated (standing and justiciability) grounds (see Burger et al. 2020, section III.C.1.b.vi), the question has yet to be tested in court, but clearly has implications beyond the specific case of *Juliana*. The purpose of this article is to review the basic principles of tort law, and assess how the science of extreme event attribution can be aligned with those principles. Our target audience is attribution scientists, as well as those working in the space between climate science and tort law. We do not address questions of standing and justiciability, which will depend on the specific legal context of the claim.

## Weyant’s core argument

In *Juliana v. United States* (2016), 21 youth plaintiffs sued the US Federal Government primarily to protect them from future harm by developing a plan to mitigate carbon release. In their suit, the plaintiffs claimed to have suffered, and would continue to suffer, “harm to their health, personal safety, bodily integrity, cultural and spiritual practices, economic stability, food security, property, and recreational interests from the impacts of climate change and ocean acidification caused by Defendants” (p. 37). The alleged impacts covered a wide range, but all were examples of the kinds of impacts that are generally expected from climate change. Both Juliana et al. and the US Government recruited expert testimony for their sides. We focus here on the core argument from the defense expert John P. Weyant (*Juliana v. United States* 2018) concerning the ability to attribute individual harms to climate change:

The reports of Plaintiffs’ experts Dr. Trenberth and Prof. Running do not and cannot reliably tie global climate change due to the Defendants’ conduct at issue to the claimed injuries they allege affected Plaintiffs . . .because the current state of our scientific understanding is not sufficiently developed. This is because: These climate and climate impact models generally cannot determine the regional effects of global climate change to the degree of specificity necessary to causally link to specific weather events, let alone to individuals and any claimed injuries.

Weyant continued:

While the local climate affects the circumstances of outcomes such as crop productivity, coastal damage from storms, frequency of wildfires, injury from heat stress, etc., *so do other factors* such as local economic growth, migration, urbanization, air and water pollution, forest and farm management processes, etc. The current set of climate and climate impact models *cannot separate* these factors with *sufficient certainty to disentangle the effect of regional climate changes* from the effect of other region-specific *confounding factors.* (emphasis added)

Weyant specifically critiqued climate scientist Kevin Trenberth’s detailing of the causes of the damage suffered by the plaintiffs in his testimony for the defense. Trenberth had used the “storyline” approach to attribution, taking the region-specific factors as contingent and assessing the role of climate change conditional on those factors. Weyant has a provocative section title in his testimony: “C. Unsupported Logical Leap of Dr. Trenberth’s Analysis”, which is followed by a claim that Trenberth had failed to make his case for the plaintiffs. Specifically, “The analytical support that Dr. Trenberth provides for each Plaintiff varies, but all his conclusions of the injuries to Plaintiffs suffer from the same failure to *connect his conditional approach conclusions to Plaintiffs’ local circumstances*.” (emphasis added).

It turns out this “logical leap” consists in the standard logical inference of *taking an instance from a generalization*: that is, Trenberth taking a general statement, generalizing over many individual cases, about a phenomenon applying to an area of the world, or a season, or a pattern of the physical world, and then picking out *one* of those cases as an example or instance of that phenomenon, which Weyant describes as an illegitimate “leap” of logic. Not even in formal logic is this deductive inference illegitimate. It is like considering a basket of red apples, taking one out of the basket without looking at it, and saying that we have a red apple. This is not fancy logic.

Nevertheless, here is the brief, bottom line version of Weyant’s “logical argument against Trenberth”, which we can analyze:

e. Dr. Trenberth states: “Plaintiff Nathan has experienced thawing permafrost and wildfires around his home in Fairbanks, Alaska, especially in 2015. Thawing permafrost is uneven and more likely on sunlight (sic) slopes, and has led to tilted and broken buildings and frost heaves in roads. Wildfires were widespread in Alaska in the summer of 2015. These harms are made worse by human-induced climate change.” As with his other examples, Dr. Trenberth does not address any *confounding factors* that might have contributed to the specific weather outcomes in Alaska in 2015, *the analysis of which is essential to reach a scientifically valid conclusion about any causal role* played by human-induced climate change on plaintiff Nathan. (emphasis added)

We would argue that “confounding factor” is not even a relevant concept here. The definition of a confounding factor is something that influences both the alleged cause and the alleged effect, introducing a spurious association that is not the causal one being alleged, and arises in the process of inductive inference (reasoning from the specific to the general). That is not the case here. We are, rather, talking about a mediating or contributing factor in what is a deductive inference. And of course we expect that the extent of impacts from climate change will depend on mediating factors such as vulnerability and exposure. There can be no frost heave in a road without a road.

## Causality in tort law

A “tort” is some act (or failing to act) that results in injury or harm to another, and amounts to a civil wrong for which courts impose liability (LII 2021). A toxic tort concerns claims of injury caused by exposure to toxins and may involve several potential defendants that brought about the exposure. Toxic tort cases resemble tort actions taken up against greenhouse gas emitters, because in both cases multiple causal factors are in play and pinning down causal responsibility and legal liability is a challenge (Burger et al. 2020).

Cranor (2005, pp. 174–177) provides a list of five steps needed to establish causality in tort law cases. We paraphrase those five steps below in italics, followed by a discussion of how they could be addressed in a case of individual climate change impacts such as *Juliana*. For this demonstration, we focus on the two harms claimed by plaintiff Nathan in the earlier quotation, namely permafrost thaw and wildfire in Alaska, but the same principles would apply to all the other claims. Note that in *Juliana*, since the assessment reports from the Intergovernmental Panel on Climate Change (IPCC) had been accepted by the US Government, its findings were taken as true. The question was then how to reason from those general findings to the specific case at issue. Given the wide acceptance of the IPCC reports, we adopt the same perspective here. Of course, depending on the jurisdiction, national assessments might also be relevant. For example, for the two harms discussed here, the chapter on Alaska in the US Fourth National Climate Assessment (Markon et al. 2018) would provide very powerful and relevant evidence. We mainly use the last comprehensive assessment report from the IPCC (the AR5), which is sufficient to make our point, because it took a consistent approach across all hazards, recognizing that there have been more recent IPCC Special Reports dealing with particular aspects of the climate system. These have generally strengthened the conclusions of the AR5.

- *First, an observed correlation or association must be shown between exposure to risk (or condition) and bad outcomes. The observed association must have an identified causal explanation, for which a responsible party should be held accountable*.

In other words, a plausible causal chain must be articulated, connecting the action of the responsible party to the bad outcome, which is reflected in observations (i.e., to what actually happened). This can be considered a scientific hypothesis. For the case of damage from permafrost thaw, for example, the causal chain would run from greenhouse gas emissions, to global warming, to regional warming, to permafrost thaw, and finally to the structural damage (e.g., buildings or roads). The first element in this chain is where the liability originates. The last step in the chain would need to be established from local experience and engineering expertise. Everything in between follows from well-established climate science. For example, according to the Technical Summary of the Working Group II report of the IPCC AR5 (IPCC 2014, Table SPM.A1, Polar regions), the observed impacts of climate change include:

Widespread permafrost degradation, especially in the southern Arctic (*high confidence*, major contribution from climate change).

In this case, as permafrost thaw is a slow onset change and is closely linked to regional warming (reflected in the high confidence level), the causal linkage is quite direct. In contrast, for the case of wildfires, the link between regional warming and wildfires is mediated by other meteorological factors such as humidity, precipitation, and windiness, as well as by non-meteorological factors such as forest management and pests, and there is also a random factor involved (ignition). Thus the IPCC attribution in this case is expressed instead as an increase in wildfire *risk or likelihood* (IPCC 2014, Table SPM.A1, North America), and is given only a medium confidence level:

Increased wildfire frequency in subarctic conifer forests and tundra (*medium confidence*, major contribution from climate change).

The establishment of causality through a chain of steps is accepted practice in climate science and climate impacts (Hegerl et al. 2010), and is especially important for connecting the science of IPCC Working Group I (physical climate) to Working Group II (impacts). However, the level of confidence in each step depends on the impact or harm being assessed (see NAS 2016).

Lusk (2017) mentions that in the now-dismissed case of *Comer v. Murphy Oil* (see Burger et al. 2020, section III.C.1.b.v), the ruling questioned the ability of the plaintiffs to attribute the increased greenhouse gas concentrations leading to the climate-change damage to the specific emissions of the defendants. From a scientific perspective, we do not see this objection as fundamentally insurmountable, since Earth System Models driven by those specific emissions could certainly be used to establish that link.

- *Second, a sufficiently complete list of possible explanations (or conditioning properties) for the bad outcomes must be enumerated*.

**Fig. 1:**
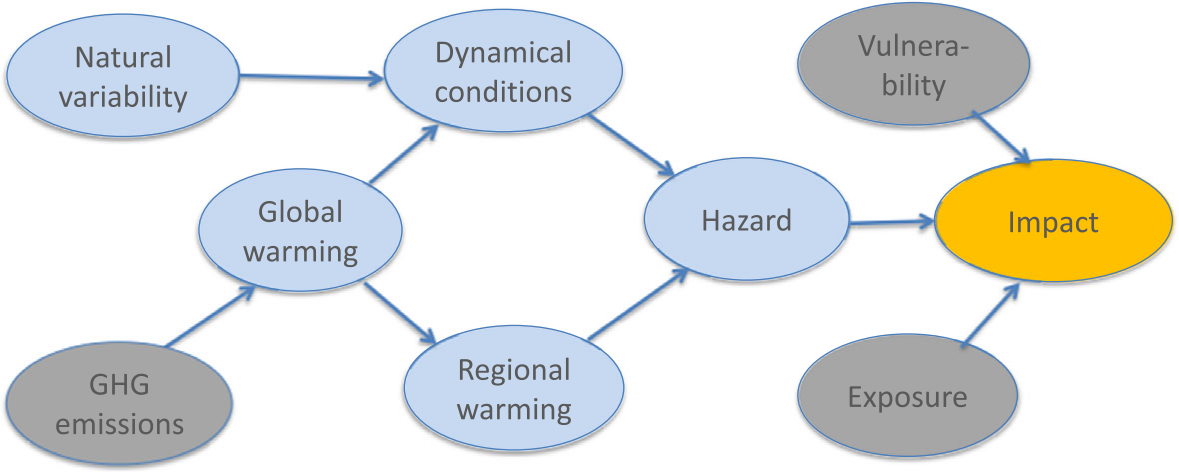
Causal graph depicting the different plausible explanations of a generic climate impact. Blue symbols denote elements in the physical climate system, gray symbols those in the human-affected domain, and the orange symbol a combination of the two. Arrows denote direction of causal influence. Note that the vulnerability may also be affected by climate change, in which case that causal influence would also need to be included

This means that as well as the primary explanation (or primary hypothesis), all plausible alternative explanations for the bad outcome must be articulated. This concept is very Bayesian: to quote Jeffreys (1961, p.58): “We get no evidence for a hypothesis by merely working out its consequences and showing that they agree with some observations, because it may happen that a wide range of other hypotheses would agree with those observations equally well. To get evidence for it we must also examine its various contradictories and show that they do not fit the observations.” In the case of climate impacts, these alternative explanations would include natural climate variability, aspects of climate change not included in the primary hypothesis, and any non-climate factors affecting the impact. The full landscape of possible explanations is depicted in a generic way in Fig. 1 (see Shepherd 2019), for the case of attribution to greenhouse gas (GHG) emissions. Here, “global warming” refers to global-mean surface temperature increase, “regional warming” refers to those aspects of climate change tied to global warming through well-understood thermodynamic mechanisms (such as Arctic amplification), and represents the primary causal explanation, while “dynamical conditions” refers to more uncertain aspects of climate change such as those related to atmospheric circulation regimes, which are strongly affected by natural variability. Permafrost thaw would be considered the “hazard” and the structural damage the “impact,” the latter being affected by both the exposure and vulnerability of those structures to the hazard. In this schema, wildfires would be considered an impact (since affected strongly by non-meteorological factors), and *wildfire risk or likelihood* (a function of various meteorological factors, e.g., Wotton 2009) the hazard. Note the vulnerability may itself be affected by climate change; e.g., the increase of pest outbreaks in boreal forests (see IPCC 2019 SPM A5.2 for high latitudes) can make those forests more prone to wildfire, in which case that causal influence would also need to be included (e.g., Figure 6 of Lloyd and Shepherd 2020).

- *Third, tests that could help discriminate between the different explanations should be considered or conducted. It is recognized that in many cases, it is infeasible to perform direct tests*.

As noted earlier, both permafrost thaw and more extensive wildfires are explainable by regional warming, which is an expected outcome of anthropogenic global warming and is expected to be especially strong in the Arctic and in Alaska in particular, as has been both observed and attributed (Taylor et al. 2017). The potential role of changing dynamical conditions and of natural variability in this regional warming could be tested through a combination of modeling and observational analysis, as is common practice in event attribution studies (Stott et al. 2016). The role of vulnerability and exposure could be tested by determining whether the appropriate local standards were adequate and were followed, e.g., construction in the case of permafrost damage, or forest management in the case of wildfire. If those standards could be shown to be inadequate or were not followed, then the liability for the bad outcomes would need to take account of those causal factors as well, which might also involve consideration of the role of climate change (e.g., whether the local standards took account of climate change). The study of van Garderen et al. (2021) has shown how the storyline approach to event attribution, implemented by constraining a climate model to follow the observed dynamical evolution of the atmosphere, is able to quantify the anthropogenic contribution to a heat wave at a high spatial and temporal resolution throughout an entire summer season. This opens the door to reliable quantification of the impact of human interventions in a particular heat-related event, if appropriate modeling tools for the impact assessment were available (e.g., a wildfire model).

- *Fourth, all relevant information must be considered in drawing a conclusion about which explanation is more likely. What constitutes relevant information for drawing a scientific conclusion is a matter of scientific judgement. One can look to consensus scientific committees for some guidance on this*.

This step is intended to reflect the fact that in scientific assessments, many different lines of evidence are used (see, e.g., Mastrandrea et al. 2011 for the IPCC guidelines). Although some lines of evidence might be considered preferable or ideal, they are not always available, and a variety of independent, individually weak lines of evidence can add up to strong evidence (again, a very Bayesian concept). In tort law cases, if useful evidence is deemed inadmissible, then it may become impossible for the plaintiffs to prove their case (Cranor 2005). The quotations from Weyant in Section 2 appear to suggest that only a fully quantitative modeling chain from the global down to the local scale, including the human-managed element, could demonstrate the required causality. However, this would be setting the bar much too high, since consensus scientific committees such as the IPCC do not require such a standard. Setting such a bar would exclude nearly all published extreme event attribution studies, which generally stop at the hazard, and do not consider the local scale. It would also exclude case studies, which by their very nature are deterministic and not expressed in terms of quantitative probabilities. Yet singular, deterministic causation is a perfectly sensible philosophical concept (Cartwright 2016). The storyline approach to extreme event attribution is essentially a case study approach, and was endorsed by a consensus scientific committee (NAS 2016). Moreover, the forensic approach to evidence used in the ecosystem expert community to understand environmental catastrophes, including wildfires, as singular events is well aligned with the storyline approach (Lloyd and Shepherd 2020).

- *Fifth, it must be shown that the bad outcome is more probable with the accountable cause than without it. This requires a consideration of alternative explanations of the observed association, including chance and confounding factors*.

The reference to “confounding factors” reflects the fact that Cranor (2005) is mainly considering the case of toxic substances, where the physical understanding of the causal linkages is generally quite weak and correlational evidence (which can be prone to the influence of confounding factors) is thus crucial. As mentioned earlier, in the case of climate impacts that are closely tied to robust consequences of global warming, the causal chain is based on physical knowledge and the reasoning is more deductive than inductive. Hence, the concept of a confounding factor is not relevant, and the issue is instead that of mediating or contributing factors. The role of non-meteorological factors has already been discussed under the third step, so we focus here on the alternative climate explanations of the hazard that led to the climate impact. In any extreme event, there is invariably a significant role played by unusual dynamical conditions, e.g., a blocking anticyclone for a heat wave. Thus, the traditional way (NAS 2016) to quantify the change in probability of a hazard due to climate change (exactly what is needed in the third step) is by the risk ratio *P*_*f*_(*H*, *D*)/*P*_*c*_(*H*, *D*), where *P* is the probability, *H* is the hazard, *D* is the dynamical condition that is conducive to that hazard, and the subscripts *f* and *c*, respectively, denote the factual conditions (with climate change) and the counterfactual conditions (without climate change). The relative roles of climate change and natural variability, or chance, are captured in the risk ratio. This calculation can sometimes be done from observations alone (using the past as a proxy for the counterfactual), but is generally done with a climate model (NAS 2016; Stott et al. 2016).

In some cases, such a calculation may be considered sufficient to prove the case. In particular, there are increasingly extreme events which scientific analysis suggests could not have happened without climate change (Burger et al. 2020). This represents “but for” (necessary) causation, which is an extremely strong form of causation. More typically, though, the analysis suggests that the event could have happened without climate change. If it was made more likely by climate change (as expressed in the risk ratio being greater than unity), this is enough to satisfy the fifth step. However, for extreme events that are strongly dynamically controlled, systematic uncertainties in the climate models may lead to an inconclusive result once those uncertainties are accounted for. If such a calculation were to be considered the only admissible form of evidence, there would be a danger that such a negative result would be interpreted as evidence against the role of climate change, even though it is widely recognized that such an inference would be logically fallacious (since absence of evidence is not evidence of absence). On the other hand, by the laws of probability, one can write (NAS 2016):

$$ \frac{P_f\left(H,D\right)}{P_c\left(H,D\right)}=\frac{P_f\left(H\ \right|\ D\Big)}{P_c\left(H\ \right|\ D\Big)}\times \frac{P_f(D)}{P_c(D)}. $$

The first factor on the right-hand side is a ratio of conditional probabilities, representing the change in probability of the hazard *for the given dynamical conditions*. Its calculation corresponds to the conditional, storyline approach discussed earlier, which quantifies what may be considered the thermodynamic aspects of change, for which there is high confidence. It can be calculated in a variety of ways, e.g., through a conditional statistical analysis (Cattiaux et al. 2010) or by constraining a climate model (van Garderen et al. 2021). Such a calculation is most naturally expressed in terms of the magnitude of the effect of climate change, and can be directly compared with the magnitude of the natural variability.

The second factor on the right-hand side of the equation represents the change in probability of the dynamical conditions due to climate change, which is not considered in the simplest form of the storyline approach. But in order for climate change to not increase the probability of the hazard, one would need to be able to argue that this second factor was strong enough to overcome the first factor. In general, this would seem to be a difficult case to make, given the well-known uncertainties surrounding the dynamical response to climate change (Shepherd 2014), and the lack of agreed-upon theories for that response. (In the absence of other knowledge, a standard Bayesian approach would be to introduce an uncertainty range in this quantity, which would be a uniform distribution centered about zero change.) In this context, it needs to be recognized that the standard of scientific proof in the tort law context is much lower (“the preponderance of evidence”, i.e., more likely than not) than the usual standard of scientific proof (Cranor 2005; Lloyd et al. 2021). In any case, were the second factor to overcome the first, that would mean that the increased hazard found in observations would be entirely attributable to natural variability. In the examples from *Juliana* of permafrost thaw and wildfire risk in Alaska discussed earlier, such a conclusion would be inconsistent with the cited IPCC attribution statements that underpinned the primary explanation in the first place.

## Discussion

The analysis presented in the previous section shows that the causal, conditional, storyline approach to extreme event attribution is well aligned with the concept of evidence that is appropriate in the legal context of tort law, which focuses on liability and causality, and may be effectively combined with probabilistic approaches which represent the more traditional approach to extreme event attribution (Stott et al. 2016). Establishing the causality of the connection between climate change and climate impacts should be much easier than in most toxic tort cases, at least for those climate impacts that are closely tied to global-mean warming, because of the clear physical understanding of the connection, and the strong science base provided by the IPCC reports. This knowledge base can be used to anchor a process of deduction, which is to say proceeding from the general to the specific, which will always be needed in any case of singular attribution. Stated colloquially, the scientific question is what known aspects of climate change tells us about extreme events, rather than what extreme events tell us about climate change (which is the usual detection and attribution perspective). That process of deduction, which can be formalized in the storyline approach, defines the primary explanation of the bad outcome, which then needs to be compared with alternative explanations in the more usual process of induction (step 5).

The principle of considering the totality of the evidence implies that in a tort law context, evidence from both the probabilistic and the more singular storyline approach to extreme event attribution should be considered, and furthermore should be set within the context of physical understanding of climate change, since the latter fundamentally underpins the reliability of extreme event attribution (NAS 2016). In other words, any particular line of evidence should not be considered in isolation. The probabilistic and storyline approaches are highly complementary in terms of their strengths and weaknesses. For example, the probabilistic approach can consider alternative aspects of climate change not considered in the storyline approach, providing an overall statistical context, and sometimes establishing “but for” causation. On the other hand, it is challenging to connect the probabilistic approach to impacts, because extreme hazard does not necessarily correspond to extreme impact (van der Wiel et al. 2020), although this issue can perhaps be mitigated to some extent if the case involves an aggregated set of impacts (Frame et al. 2020). If the probabilistic approach suggests that the known thermodynamic aspects of change are the main drivers of change in hazard (as is often the case), then the storyline approach could be used to more reliably quantify impacts and harm, since it does not need to blur over what might be essential details of the event in order to create a sample population for the probability estimates. In this way, the storyline approach can establish deterministic “but for” causation, albeit in a conditional manner.

An example of this sort of synergy is illustrated by two recent studies concerning the glacier lake outburst flood risk from Lake Palcacocha to the downstream city of Huarez, Peru. This is the subject of the case *Lliuya v. RWE AG* where a Peruvian farmer filed suit in German court against a German utility company, arguing that the utility’s greenhouse gas emissions have increased the risk (see Burger et al. 2020, section III.C.5.c.iii). (The case sought only the percentage of costs corresponding to RWE’s contribution to total global industrial greenhouse gas emissions, estimated at 0.5%. The case is still pending, having been delayed by COVID-19.) Stuart-Smith et al. (2021) performed a probabilistic assessment using a detailed glacier mass-balance model to show that the observed glacier retreat is as likely as not to have been entirely driven by anthropogenic greenhouse gas emissions, and virtually certain to have not arisen from natural variability. Note the glacier retreat and rising lake levels may be considered a slow-onset aspect of climate change, thus not an extreme event per se. They then argued that the glacier lake outburst flood risk had thereby increased. Huggel et al. (2020) instead took a storyline approach, developing a causal chain (analogous to Fig. 1, but elaborated for this particular impact) and discussing the evidence for each of the causal linkages, and the potential role of various confounding factors (i.e., alternative explanations). In this way, they created an evidentiary narrative. Huggel et al. also discussed in detail the complex institutional and cultural factors that shape the actual risk within Huarez itself. These two studies are clearly highly complementary and together span the causal chain from global to local.

Our analysis suggests that in order to effectively support climate change litigation, extreme event attribution studies should be framed and conducted in such a way that they can be used in conjunction with other studies to build an overall evidentiary narrative embedded within a causal framework, as in Fig. 1. This would seem to call for the complementary development of storyline and probabilistic approaches to attribution. The connection to detailed impacts at the local scale is most readily achieved with the storyline approach, which can provide a conditional explanation of the observed event, and permit a reliable quantification of the impact of the various causal factors, including exposure and vulnerability. The latter may combine with the hazard in a highly nonlinear fashion that is very sensitive to the event definition, making the storyline approach particularly compelling (as there is very little freedom in the event definition, and little need for downscaling or bias correction if suitable data is available to track the event). Probabilistic event attribution, or other evidence (such as observed trends), can then address the question of alternative explanations, such as whether the conditional aspects of the storyline calculation (namely the dynamical conditions leading to the event) might themselves respond to climate change, and need to be accounted for in the explanation. This requires that probabilistic attribution studies not state their results simply as an unconditional attribution to climate change alone, but partition the attribution into thermodynamic and dynamical factors, and discuss the potential attribution of each to climate change. (This was indeed one of the recommendations of NAS (2016).) For example, in a recent attribution study of the 2019 Australian wildfires, van Oldenborgh et al. (2021) considered the different drivers of circulation anomalies that increased wildfire risk that year, and concluded that there was no evidence that any of them had been affected by climate change. That entirely thermodynamic explanation of the probabilistic climate attribution opens the door to a storyline analysis of that wildfire season, using the details of the meteorological conditions as they actually occurred rather than an abstract event class which is not of a matching geographic and temporal scope to the actual event.

We return to the statement from Burger et al. (2020) at the beginning of this paper, which posed the question of “to what extent observational evidence of local impacts … can be used to support claims of injury in the absence of an attribution study of a matching geographic and temporal scope”. Burger et al. (2020) go on to say:

The answer to this question of course depends on context, but generally speaking, such observational evidence should be interpreted in light of the larger body of attribution research and assigned weight accordingly. For example, if plaintiffs submit evidence that anthropogenic influence on climate is driving snowpack declines throughout the Northern Hemisphere, then it would be reasonable to infer that the observed declines in snowpack at particular resorts in North America have also been caused by anthropogenic influence on climate even without a radically downscaled attribution study for those resorts.

This is exactly the deductive process of arguing from the general to the specific. In Section 3, we have shown how this can be carried forward for each of Cranor’s five steps.

Often the most important fight over scientific evidence occurs in the pre-trial process. Cranor (2005) emphasizes how important it is that decisions on the admissibility of scientific evidence not set the bar too high, and require a standard of scientific proof that exceeds that used by the scientific community itself. In particular, we have shown here that Weyant’s claim in the *Juliana* case that we cannot reach a “scientifically valid conclusion about *any* causal role played by human-induced climate change” (emphasis added) on the plaintiffs is, quite simply, scientifically incorrect. Because legal decisions can cast long shadows (Cranor 2005), the climate science community needs to be vigilant in how its knowledge is used (or dismissed) in the legal context.
